# Factor analysis of lifetime psychopathology and its brain morphometric and genetic correlates in a transdiagnostic sample

**DOI:** 10.1038/s41398-024-02936-6

**Published:** 2024-06-03

**Authors:** Axel Krug, Frederike Stein, Friederike S. David, Simon Schmitt, Katharina Brosch, Julia-Katharina Pfarr, Kai G. Ringwald, Tina Meller, Florian Thomas-Odenthal, Susanne Meinert, Katharina Thiel, Alexandra Winter, Lena Waltemate, Hannah Lemke, Dominik Grotegerd, Nils Opel, Jonathan Repple, Tim Hahn, Fabian Streit, Stephanie H. Witt, Marcella Rietschel, Till F. M. Andlauer, Markus M. Nöthen, Alexandra Philipsen, Igor Nenadić, Udo Dannlowski, Tilo Kircher, Andreas J. Forstner

**Affiliations:** 1 Department of Psychiatry and Psychotherapy, University Hospital Bonn, Bonn, Germany; 2 Department of Psychiatry and Psychotherapy, University of Marburg, Marburg, Germany; 3 Center for Mind, Brain and Behavior, University of Marburg, Marburg, Germany; 4 Institute of Human Genetics, University of Bonn, School of Medicine & University Hospital Bonn, Bonn, Germany; 5 Department of Psychiatry, Social Psychiatry and Psychotherapy, Hannover Medical School, Hannover, Germany; 6 Institute of Behavioral Science, Feinstein Institutes for Medical Research, Manhasset, NY, USA; 7 Institute for Translational Psychiatry, University of Münster, Münster, Germany; 8 Institute for Translational Neuroscience, University of Münster, Münster, Germany; 9 German Centre for Mental Health (DZPG), Site Jena-Magdeburg-Halle, Jena, Germany; 10 Department of Psychiatry and Psychotherapy, University Hospital Jena, Jena, Germany; 11 Goethe University Frankfurt, University Hospital, Department of Psychiatry, Psychosomatic Medicine and Psychotherapy, Frankfurt, Germany; 12 Department of Genetic Epidemiology in Psychiatry, Central Institute of Mental Health, Medical Faculty Mannheim, Heidelberg University, Mannheim, Germany; 13 Department of Psychiatry and Psychotherapy, Central Institute of Mental Health, Medical Faculty Mannheim, Heidelberg University, Mannheim, Germany; 14 Hector Institute for Artificial Intelligence in Psychiatry, Central Institute of Mental Health, Medical Faculty Mannheim, Heidelberg University, Mannheim, Germany; 15 Central Institute of Mental Health, Medical Faculty Mannheim, Heidelberg University, J5, 68159 Mannheim, Germany; 16 Department of Neurology, Department of Neurology, Klinikum rechts der Isar, Technical University of Munich, Munich, Germany; 17 Institute of Neuroscience and Medicine (INM-1), Research Center Jülich, Jülich, Germany; 18 Centre for Human Genetics, University of Marburg, Marburg, Germany

**Keywords:** Schizophrenia, Clinical genetics, Molecular neuroscience

## Abstract

There is a lack of knowledge regarding the relationship between proneness to dimensional psychopathological syndromes and the underlying pathogenesis across major psychiatric disorders, i.e., Major Depressive Disorder (MDD), Bipolar Disorder (BD), Schizoaffective Disorder (SZA), and Schizophrenia (SZ). Lifetime psychopathology was assessed using the OPerational CRITeria (OPCRIT) system in 1,038 patients meeting DSM-IV-TR criteria for MDD, BD, SZ, or SZA. The cohort was split into two samples for exploratory and confirmatory factor analyses. All patients were scanned with 3-T MRI, and data was analyzed with the CAT-12 toolbox in SPM12. Psychopathological factor scores were correlated with gray matter volume (GMV) and cortical thickness (CT). Finally, factor scores were used for exploratory genetic analyses including genome-wide association studies (GWAS) and polygenic risk score (PRS) association analyses. Three factors (paranoid-hallucinatory syndrome, PHS; mania, MA; depression, DEP) were identified and cross-validated. PHS was negatively correlated with four GMV clusters comprising parts of the hippocampus, amygdala, angular, middle occipital, and middle frontal gyri. PHS was also negatively associated with the bilateral superior temporal, left parietal operculum, and right angular gyrus CT. No significant brain correlates were observed for the two other psychopathological factors. We identified genome-wide significant associations for MA and DEP. PRS for MDD and SZ showed a positive effect on PHS, while PRS for BD showed a positive effect on all three factors. This study investigated the relationship of lifetime psychopathological factors and brain morphometric and genetic markers. Results highlight the need for dimensional approaches, overcoming the limitations of the current psychiatric nosology.

## Introduction

There is a long tradition of investigating the relationship between psychopathological syndromes and brain structure and function in patients suffering from schizophrenia (SZ) and schizoaffective disorder – henceforth referred to as schizophrenia spectrum disorders (SSD), as well as bipolar disorder (BD), and major depressive disorder (MDD). Several studies have linked specific symptoms such as verbal hallucinations to local brain structures, particularly the bilateral superior temporal gyri [1–3]. However, these have been either low in statistical power or variance [4], or limited to a specific diagnosis, such as SZ [5, 6]. This raises the question of generalizability across diagnostic categories: Since almost all symptoms can be present in different diagnoses (e.g., formal thought disorders are found in SZ, as well as in BD, and in MDD) [7–9], it is of major interest to study these syndromes transdiagnostically using dimensional approaches. Moreover, the phenotypic overlap between psychiatric disorders is also reflected at a brain structural [10–13] as well as genetic level [14].

Factor analyses of lifetime psychopathology have mostly been performed within one categorical disorder. Only a few studies are available, investigating transdiagnostic symptom dimensions of lifetime psychopathology across diagnoses: Investigating patients with DSM-IV diagnosed SZ, BD and delusional disorder, Serretti and Olgiati found that a five-factor model best described lifetime symptom dimensions [15]. In a sample consisting of patients with SZ, BD, MDD, delusional, and psychotic disorder not otherwise specified, a four-factor solution was obtained, consisting of excitement, psychotic features (hallucinations and delusions), depression and disorganization [16]. Studying the factor structure of the OPerational CRITeria (OPCRIT) system in the SZ spectrum and BD, Reininghaus and colleagues obtained a bifactor model with one transdiagnostic psychosis dimension and five specific factors comprising positive, negative, manic, disorganized and depressive symptoms [17].

Previously, most structural and functional magnetic resonance neuroimaging studies focused on categorical comparisons of one patient group (MDD, BD, or SSD) compared to a healthy control (HC) group. However, these studies failed to identify structural and functional brain correlates that separate disorders [18]. In contrast, studies and meta-analyses indicated common alterations across diagnoses [11–13, 19, 20]. Transdiagnostic studies of dimensional psychopathology might thus be more promising regarding identification of common risk factors and might especially lead to a more precise treatment of these syndromes on a transdiagnostic rather than diagnosis-based level. In addition, they should be able to take into account the heterogeneity of psychiatric disorders as well as potential comorbidities. This should also help to identify specific neurobiological markers which in turn can inform personalized treatment interventions.

Twin and family studies demonstrate that genetic factors contribute substantially to the development of MDD, BD and SZ, with heritability estimates of around 60% to 85% for SZ and BD [21–23] and around 40% for MDD [24]. Recent genome-wide association studies (GWAS) have identified numerous genome-wide significant loci for all three psychiatric disorders (e.g., refs. [25, 26]). Furthermore, transdiagnostic GWAS meta-analyses have demonstrated an extensive genetic overlap between MDD, BD and SZ [14]. Byrne et al. provided evidence that only a small subset of the genome-wide significant variants for SZ and MDD have disorder-specific effects [27]. One plausible hypothesis, therefore, is that pleiotropic genetic variants mediate their disease risk via effects on transdiagnostic symptom dimensions. In addition, an analysis of polygenic risk scores (PRS), which summarize the effects of multiple common genetic variants into an individual genetic risk profile [28], by Ruderfer et al. showed that the PRS for SZ was significantly increased in BD patients with psychotic features and SZ patients with prominent negative symptoms [29]. These results suggest that there are genetic factors underlying specific symptom dimensions within both disorders [29].

As symptom presentations can fluctuate within an individual patient over the course of life and even within a single episode, the aim of the present study was to i) assess lifetime symptoms in a transdiagnostic sample to identify underlying symptom factors; and ii) investigate the relationship of detected factors with local GMV and CT. Considering that brain structure is less variable within a short period of time, we hypothesize that this approach would yield more conclusive results than correlating GMV with psychopathology present at any given point in time. In addition, applying both GMV as well as CT measures should render a fuller picture of underlying mechanisms as we would not assume that all potential associations would be based on one measure alone. Finally, iii) it was explored if the detected factor structure can be linked to common genetic variation. Based on previous brain-morphometric and genetic studies, we hypothesized findings from specific DSM-IV diagnostic categories to be present across diagnoses, too.

## Material and methods

### Participants

Patients were recruited as part of the FOR2107 cohort [30] (www.for2107.de). Patient recruitment took place via the in-patient facilities of the University hospitals in Marburg and Münster, Germany, through participating hospitals, and via postings in local newspapers. Written informed consent was obtained from all patients before participation. According to the Declaration of Helsinki, all procedures were approved by the local ethics committees. After study participation, all patients received financial compensation. After excluding patients with incomplete data, serious medical illnesses, neurological illnesses, and an IQ < 80, we analyzed a total of *N* = 1038 patients (see Table 1a, b, required sample size is based on [31]) suffering from MDD, BD, and SSD (aged 18–65).

**Table 1:** a: Characteristics of the explorative sample *n* = 520. b: Characteristics of the confirmatory sample *n* = 518.

	Major depressive disorder (*n* = 402)	Bipolar disorder (*n* = 64)	Schizophrenia spectrum disorders (*n* = 54)	Group comparison (F/Chi-values in brackets)
**a**				
Age	36.53 (13.45)	41.05 (11.96)	37.46 (11.75)	*p* = 0.005^a^ (5.34)
Sex	*m* = 140 *f* = 262	*m* = 30 *f* = 34	*m* = 28 *f* = 26	*p* = 0.016 (8.15)
Years of education	13.25 (2.68)	13.5 (2.79)	12.54 (2.45)	*p* = 0.213 (1.55)
Age of onset	26.14 (12.64)	21.97 (10.66)	22.54 (10.26)	*p* = 0.034^b^ (3.4)
TIV	1563.7 (160.75)	1578.16 (149.26)	1580.85 (152.16)	*p* = 0.685 (0.38)

	Major depressive disorder (*n* = 401)	Bipolar disorder (*n* = 63)	Schizophrenia spectrums disorders (*n* = 54)	Group comparison (F/Chi-values in brackets)
**b**				
Age	36.53 (12.84)	41.03 (12.31)	37.48 (11.27)	*p* = 0.023^a^ (3.8)
Sex	*m* = 140 *f* = 261	*m* = 30 *f* = 33	*m* = 29 *f* = 25	*p* = 0.008 (9.67)
Years of education	13.25 (2.79)	14.47 (2.8)	12.51 (2.84)	*p* = 0.001^b^ (1.55)
Age of onset	25.52 (12.28)	26.0 (11.18)	22.89 (8.27)	*p* = 0.23 (1.48)
TIV	1558.8 (148.18)	1596.85 (134.22)	1584.76 (206.19)	*p* = 0.193 (1.65)

*TIV* total intracranial volume.

^a^MDD < BD.

^b^MDD < BD; SSD < BD.

### Psychopathological assessment and factor score calculation

The German version of the structured interview SCID-I (DSM-IV-TR [32]) and the OPCRIT (version 4 [33]) were administered in all patients. Lifetime psychopathology was assessed as any occurrence of symptoms during the life span until data acquisition. Trained personnel assessed lifetime symptoms based on patients’ reports and additional hospital records, when available. Numerous interview trainings assured data quality. Interrater-reliability was assessed with the interclass coefficient, achieving good reliability of *r* > 0.86. For the present study, only symptomatic items were included (items 17–77). Following the procedures described in Stein et al. [34], we separated the total cohort (*N* = 1038) into two samples using the “mindiff” [35] package in R [36] accounting for age, sex, and diagnostic category (i.e., same distribution of categorical diagnoses across both samples). In the first sample of *n* = 520, we performed varimax rotated principal axis factor analyses with bootstrapping (5000 permutations) using the *psych* [37] and *EFAutilities* [38] packages in R (v4.0.5.) for models with 2–5 factors. Hereof, z-transformed values were used since the data was differently scaled. For interpretation purposes, items with factor loadings <0.5 were not considered in the analysis [34]. Cronbach’s alpha coefficients [39] were used to test the internal consistency of the explorative factors. Using the second sample of *n* = 518, we cross-validated the explorative models using confirmatory factor analysis in Mplus (version 8.4 [40]). Confirmatory model estimation was performed using the maximum-likelihood-method (MLM) since this estimator is robust to standard errors and is one of the most common estimators [41]. The following fit indices were used: chi-square significance test, comparative fit index (CFI [42]) and Root Mean Square Error of Approximation (RMSEA [43]). Based on these fit indices, we evaluated the different models and selected the one with best fit. After cross-validating the explorative model in the second sample, we tested the model for the whole sample (*N* = 1038).

As the DSM-IV-TR diagnostic groups were unequally distributed, we wanted to rule out potential confounding effects of formal diagnosis. Therefore, we tested the selected factorial model in an age- and sex-matched sample with an equal diagnosis distribution (each *n* = 108 of MDD, BD, SSD, total *n* = 324) (see supplement eTable1). Matching was performed using the “MatchIt” package [44] in R [36]. Furthermore, the factorial model was also tested within each of the three diagnostic categories and factor loadings were compared between DSM diagnosis using non-parametric Kruskal–Wallis tests (see supplement).

### MRI assessment and preprocessing

Subjects were scanned with a 3-T MRI at the Department of Psychiatry and Psychotherapy in Marburg (Tim Trio, Siemens, Erlangen, Germany; 12-channel head coil) and the Institute for Translational Psychiatry in Münster (Prisma, Siemens, Erlangen, Germany; 20-channel head coil). MRI data were acquired according to an extensive quality assurance protocol [45]. A fast gradient echo MP-RAGE sequence with a slice thickness of 1.0 mm consisting of 176 sagittal orientated slices in Marburg and 192 in Münster and a FOV of 256 mm was used to acquire T1 weighted images. Parameters differed across sites: Marburg: TR = 1.9 s, TE = 2.26 ms, TI = 900 ms, flip angle = 9°; Münster: TR = 2.13 s, TE = 2.28 ms, TI = 900 ms, flip angle = 8°.

For a detailed description of the preprocessing of MRI data please see refs. [31, 46]. In short, both voxel-based-morphometry GMV and cortical thickness (CT) data were preprocessed using the default parameters as implemented in the CAT12-Toolbox (Computation Anatomy Toolbox for SPM, build 1184, Structural Brain Mapping group, Jena University Hospital, Germany) building on SPM12 (Statistical Parametric Mapping, Institute of Neurology, London, UK). We opted for GMV and CT over other MRI-derived metrics for two primary reasons. Firstly, volume and thickness measures, commonly employed in large-scale analyses such as those by the ENIGMA consortium, were selected to facilitate result comparisons. Second, recent neuroimaging research has underscored the complementary nature of GMV and CT measurements. GMV provides insight into overall gray matter volume, which can reflect global brain atrophy or neurodevelopmental factors. In contrast, CT offers information about the thickness of the cortical mantle, allowing for the detection of localized changes. By analyzing both GMV and CT, we aimed to capture both global and local structural alterations in the context of these psychiatric disorders [47, 48]. Images were spatially registered, segmented [49] and normalized [50]. CT preprocessing included fully-automated methods projecting local maxima to other GM voxels using a neighbor relationship described by the white matter distance [51]. Quality control of processed data was performed as implemented in CAT12. For GMV data, a Gaussian kernel of 8 mm FWHM was used for smoothing. For CT data, a Gaussian kernel of 20 mm FWHM was used.

### Statistical analyses: gray matter volume and cortical thickness

For both GMV and CT analyses, we used separate linear regression models for each factor using CAT12 and SPM12. The following nuisance variables were included in brain structural analyses: age, sex, and two dummy-coded variables accounting for the different MRI scanners and a body coil exchange in Marburg (Marburg pre body coil: yes/no, Marburg post body coil: yes/no, Münster as reference category [30, 45]). To control for potential medication effects, we used three dummy coded (yes/no) covariates accounting for the current medication with antidepressants, mood stabilizers and antipsychotics. For GMV analyses total intracranial volume was additionally accounted as covariate of no interest and absolute threshold masking with a threshold value of 0.1 was used.

To further test confounding effects of unequally distributed diagnostic categories, we performed multiple regression analyses in the age and sex matched sample (*n* = 324) with same n per diagnosis, again. Besides this whole brain analysis, we additionally performed ROI analyses of the detected clusters from the total sample in the matched sub-sample (see supplement).

In addition to multiple regression analyses, we performed full factorial ANCOVA whole brain interaction analyses (factor x diagnosis) for each of the three factors to test whether transdiagnostic brain correlates were driven by single DSM-IV-TR diagnostic categories for both the total and the matched sample with same n per diagnosis. Moreover, post hoc interaction analyses (factor x diagnosis) were performed specifically within each detected cluster of the total sample using the “lm-function” in R.

Cluster labeling was applied using the dartel space Neuromorphometrics atlas (http://www.neuromorphometrics.com/) for GMV analyses and for CT analyses using the Desikan–Killiany atlas [52]. Results were suggested significant at *p* < 0.05 peak-level, family wise error (FWE) corrected, cluster extend *k* = 35 voxels in the total and matched sample.

### GWAS and PRS association analysis

DNA extraction, genome-wide genotyping, quality control and imputation were carried out as previously described [53] in the full FOR2107 cohort. Briefly, genotyping was performed using genomic DNA from blood samples and the Infinium PsychArray BeadChip (Illumina, San Diego, CA, USA). Pre-imputation quality control (QC) was performed in GenomeStudio, PLINK v1.9 [54], and R [36], with removal of genetic variants and individuals according to standard filter criteria. Genotype data were imputed to the 1000 Genomes phase 3 reference panel [55] using SHAPEIT [56] and IMPUTE2 [57]. In post-imputation QC, variants with a minor allele frequency <1%, Hardy-Weinberg equilibrium *p* < 1e−6, and an INFO metric <0.8 were removed. From the total sample of the present study (*N* = 1038), high-quality genotype data were available for 951 individuals. From these, 13 individuals were excluded due to intra-sample relatedness (π ^ > 0.125), resulting in a sample of *n* = 938 individuals used for genetic analyses.

For each of the three factors, GWAS, which should be considered exploratory at the given sample size, were conducted via linear regression in PLINK with rank-based inverse normal transformed values [58] as quantitative phenotypes due to the non-normal distribution of factor scores. Sex, age and the first four multidimensional scaling (MDS) components were included as covariates. All variables were z-scaled via the ‘standard-beta’ modifier for better comparability between factor dimensions. We performed clumping of genetic markers in the GWAS results using a maximum *p* value of 1e−4 for index variants (‘--clump-p1 1e−4’), an LD threshold of 0.1 (‘--clump-r2 0.1’), and a window size of 1000 kb (‘--clump-kb 500’). We considered genetic associations with *p* < 5e−8 to be genome-wide significant and with *p* < 1e−6 to be suggestive. We performed gene-based and gene-set analyses with MAGMA [59] as implemented in FUMA [60]. The resulting *p* values were corrected for multiple testing using the Bonferroni method taking into account the number of tested genes (*n* = 18,846) or gene sets (*n* = 10,678). We used LocusZoom [61] to generate regional plots.

PRS for MDD, BD and SZ were calculated based on publicly available summary statistics from three studies [25, 26, 62]. Variant weights for PRS calculation were estimated with PRS-CS [63] using default parameters and a set of pre-defined values for the global shrinkage parameter φ (1e−4, 1e−3, 1e−2). PRS were subsequently calculated in R [36] as described previously [64]. Linear additive models with rank-based inverse normal transformed factor scores as outcome, one of the z-scaled disorder-specific PRS as predictor and sex, age and the first four MDS components as covariates were fitted in R. The PRS association analysis was conducted for both the complete set of *n* = 938 individuals as well as for each diagnostic subgroup separately. Adjustment of *p* values for multiple testing was performed with the Benjamini–Hochberg approach [65] within each set of 27 tests (3 factor dimensions * 3 PRS models * 3 values for φ). Model coefficients were considered to be statistically significant at *p* < 0.05. We calculated the variance explained (R^2^) by each PRS as the difference between R^2^ of the full model and R^2^ of the null model containing only the covariates.

## Results

### Exploratory and confirmatory factor analyses

We tested explorative models ranging from 2-5 factors. Results of these models can be found in Supplementary eTables 2a–d. In a next step, we evaluated the four explorative models using confirmatory analyses in the second sample. Model fits were as follows: a) 2 factors: *χ*^*2*^ = 393.645, *df* = 224, *p* < 0.001, *CFI* = 0.903, *RMSEA* = 0.038; b) 3 factors: *χ*^*2*^ = 543.005, *df* = 316, *p* < 0.001, *CFI* = 0.904, *RMSEA* = 0.037; c) 4 factors: *χ*^*2*^ = 588.773, *df* = 314, *p* < 0.001, *CFI* = 0.875, *RMSEA* = 0.042; d) 5 factors: *χ*^*2*^ = 748.705, *df* = 391, *p* < 0.001, *CFI* = 0.884, *RMSEA* = 0.041. Based on the fit indices, we decided to use model b) with 3 factors (Table 2) for further analyses as this model outperformed the other ones. Moreover, a 3-factor model is also in line with the Scree Plot (Supplementary eFigure 1). The model included the factors paranoid-hallucinatory syndrome (PHS) (explaining 14% of total variance), mania (MA) (explaining 11% of total variance), and depression (DEP) (explaining 5% of total variance). Furthermore, we performed a confirmatory factor analysis in the whole sample (*N* = 1038) showing a good fit *χ*^*2*^ = 605.667, *df* = 316, *p* < 0.0001, *CFI* = 0.932, *RMSEA* = 0.03. Results of the confirmatory analyses of the matched sample and within each diagnostic category are presented in the supplement (Supplementary eResults1 and 2). We investigated differences of the factor loadings between diagnostic categories using a non-parametric ANOVA (Kruskal–Wallis). Diagnostic groups differed significantly in all three factors identified (Supplementary eResults3 and Supplementary eFigure 2).

**Table 2:** Explorative factor model of sample 1, *n* = 520.

Factor	Item	Symptom	Loading	Cronbach´s Alpha
1 (PHS)	Opcrit61	Delusions of passivity	0.711	0.910
	Opcrit64	Delusions and hallucinations last for one week	0.700	
	Opcrit68	Thought broadcast	0.697	
	Opcrit66	Thought insertion	0.681	
	Opcrit58	Delusions of influence	0.681	
	Opcrit62	Primary delusional perception	0.657	
	Opcrit55	Well organized delusions	0.650	
	Opcrit60	Widespread delusions	0.621	
	Opcrit54	Persecutory delusions	0.620	
	Opcrit74	Running commentary voices	0.602	
	Opcrit73	Third person auditory hallucinations	0.571	
	Opcrit59	Bizarre delusions	0.552	
	Opcrit67	Thought withdrawal	0.549	
	Opcrit63	Other primary delusions	0.543	
	Opcrit77	Non-affective hallucination in any modality	0.522	
2 (MA)	Opcrit35	Elevated mood	0.890	0.921
	Opcrit19	Excessive activity	0.829	
	Opcrit30	Pressured speech	0.804	
	Opcrit56	Increased self esteem	0.795	
	Opcrit20	Reckless activity	0.765	
	Opcrit22	Reduced need for sleep	0.760	
	Opcrit31	Thoughts racing	0.732	
	Opcrit21	Distractibility	0.525	
3 (DEP)	Opcrit39	Loss of pleasure	0.678	0.736
	Opcrit25	Loss of energy/tiredness	0.635	
	Opcrit37	Dysphoria	0.603	
	Opcrit41	Lack of concentration	0.507	

### Brain morphometric correlates of life-time psychopathology

**Fig. 1:**
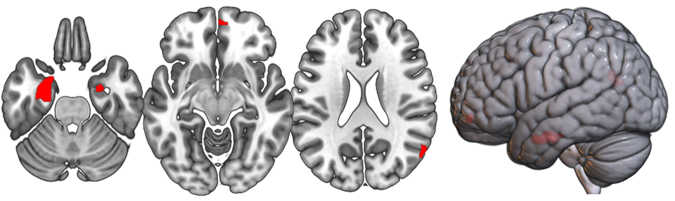
Local GMV correlates of the lifetime paranoid-hallucinatory syndrome (PHS). Negative association of factor 1 paranoid-hallucinatory syndrome (PHS) and gray matter volume (GMV) comprising parts of the bilateral hippocampus, amygdala, and right angular gyrus across patients with major depressive disorder, bipolar disorder, and schizophrenia spectrum disorders. Clusters are shown at *p* < 0.05 peak-level, family-wise error-corrected.

**Fig. 2:**
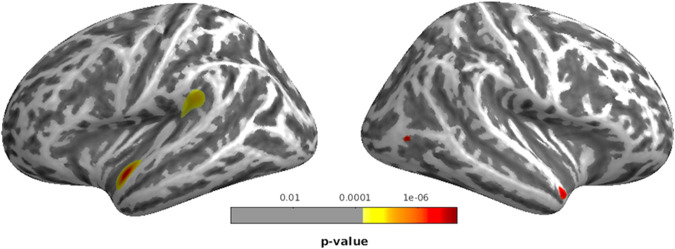
CT correlates of the paranoid-hallucinatory syndrome (PHS). Negative association of factor 1 paranoid-hallucinatory syndrome (PHS) and cortical thickness (CT) comprising parts of left supramariginal, bilateral superior temporal, and right lateral occipital clusters across patients with major depressive disorder, bipolar disorder, and schizophrenia spectrum disorders. Clusters are shown at *p* < 0.05 peak-level, family-wise error-corrected.

Results of the multiple regression analyses of the total sample are displayed in Table 3 (GMV) and 4 (CT). For the paranoid-hallucinatory syndrome (PHS), negative GMV correlations were observed in the bilateral hippocampus, amygdala, and right angular gyrus (see Fig. 1). CT was negatively correlated with the paranoid-hallucinatory syndrome (PHS) comprising left supramariginal, bilateral superior temporal, and right lateral occipital clusters (see Fig. 2). Whole-brain interaction analyses revealed no significant interaction of psychopathological factor and DSM-IV-TR diagnosis for both GMV and CT. Post hoc interaction analyses on the significant clusters in Tables 3 and 4 revealed no significant interactions of factor x diagnosis (all *p*s > 0.05, see Supplementary eResults4 and Supplementary eFig. 3–10 for details). Results of the GMV and CT analyses in the matched sample are presented in the supplement (Supplementary eResults 5, Supplementary eTables 3 and 4). Here, results from the total sample could be replicated. We did not find any associations with the DEP and MA factors for both GMV and CT.

**Table 3:** Results of the lifetime paranoid-hallucinatory syndrome (PHS) and its local gray matter (GMV) correlates.

		MNI coordinates				
Anatomical region	H	X	Y	Z	t-value	Cluster size
Factor I: Paranoid-hallucinatory syndrome: gray matter volume						
Entorhinal area, hippocampus, amygdala, parahippocampal gyrus, fusiform gyrus, temporal pole	L	−27	−8	−27	5.53	560
Angular gyrus, middle occipital gyrus	R	57	−64.5	22.5	5.13	150
Amygdala, hippocampus, entorhinal area	R	25.5	−4.5	−27	5.00	83
Medial frontal cerebrum	R	3	57	−10.5	4.81	64

Only negative correlations are reported as no positive correlations were detected.

*H* hemisphere, *L* left, *R* right.

**Table 4:** Results of the lifetime paranoid-hallucinatory syndrome (PHS) and its cortical thickness correlates.

	MNI Coordinates						
Anatomical region	H	X	Y	Z		t-value	Cluster size
Factor I: Paranoid-hallucinatory syndrome: Cortical thickness							
Superior temporal cortex	L	−48.3	−3.62	−12.1		5.07	657
Supramarginal cortex	L	−48.3	−38.5	25.9		4.34	777
Superior temporal cortex	R	48.6	15.6	−22.6		4.11	236
Lateral occipital cortex	R	44.6	−71.7	3.1		4.00	47

Only negative correlations are reported as no positive correlations were detected.

*H* hemisphere, *L* left, *R* right.

### Genetic correlates of life-time psychopathology

**Fig. 3:**
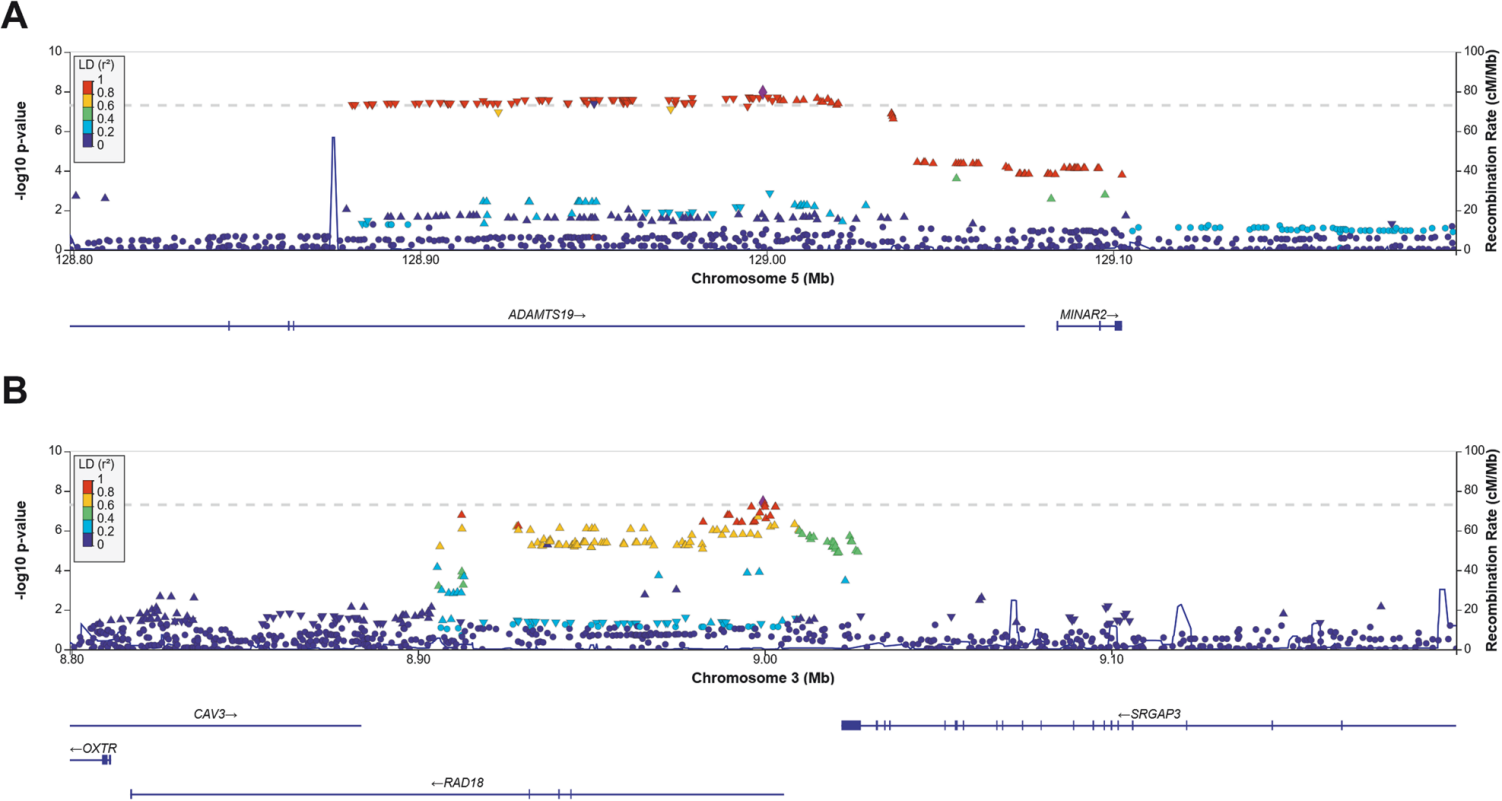
Genetic loci with genome-wide significant association. Regional plots with a window size of 500 kb are shown for the genome-wide significant associations with MA “mania” (**A**), and DEP “depression” (**B**). The respective lead variants rs10062519 and rs11131155 are depicted as linkage disequilibrium reference variants (purple diamonds). cM centimorgan, LD Ref Var, linkage disequilibrium reference variant, Mb megabase.

Our exploratory GWAS revealed genome-wide significant associations for MA and DEP (Fig. 3, Supplementary eFigs. 11–13, Supplementary eTable 5), with intronic lead variants rs10062519 (*p* = 1.10e−8) located in *ADAMTS19* for MA and rs11131155 (*p* = 4.12e−8) located in *RAD18* for DEP. In the MAGMA gene analysis, a genome-wide significant association was identified for *SYTL1* (DEP, *p* = 1.79e−6). The MAGMA gene-set analysis yielded no statistically significant results for any of the three factors after correction for multiple testing (data not shown).

**Fig. 4:**
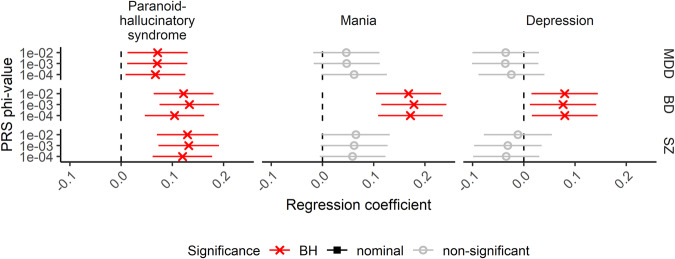
PRS association analysis. Regression of the three factors on the PRS for MDD, BD, and SZ shows significant effects of PRS for MDD, BD, and SZ on PHS “paranoid-hallucinatory syndrome” and of PRS for BD on MA “mania” and DEP “depression” in the full transdiagnostic sample. BD bipolar disorder, BH Benjamini–Hochberg, MDD major depressive disorder, SZ schizophrenia.

In the PRS association analysis of the complete sample (Fig. 4), we detected a positive effect of PRS for BD on all three factors (PHS: maximum *β* = 0.13 at φ = 1e−3 with *R*^*2*^ = 0.021 and adjusted *p* = 5.48e−5; MA: maximum *β* = 0.18 at φ = 1e−3 with *R*^*2*^ = 0.031 and adjusted *p* = 1.17e−6; DEP: maximum *β* = 0.08 at φ = 1e−4 with *R*^*2*^ = 0.006 and adjusted *p* = 0.038). Further, a positive effect on PHS was observed for the PRS for MDD (maximum *β* = 0.07 at φ = 1e−2 with *R*^*2*^ = 0.006 and adjusted *p* = 0.038) and SZ (maximum *β* = 0.13 at φ = 1e−3 with *R*^*2*^ = 0.020 and adjusted *p* = 7.06e−5). In the subset analysis of each diagnostic group, none of the effects observed in the complete transdiagnostic sample reached statistical significance (Supplementary eFig. 14).

## Discussion

In the present study, exploratory and confirmatory factor analyses of lifetime psychopathology revealed a three-factor model with superior fit properties compared to models with less or more factors. Factors were the paranoid-hallucinatory syndrome (PHS), mania (MA) and depression (DEP). In addition, several associations with both brain morphometry and genetics were reported. This study represents a successful advancement of previous research by Stein et al. [34] and David et al. [66] all of them part of the FOR2107 cohort, wherein five factors of acute psychopathology were described and genetically investigated.

Compared to previous factor analytical research in the three diagnoses included in our study, utilizing the OPCRIT, the present factor solution features three factors, while other studies showed an additional negative factor, which was not present in our study. Nevertheless, an overlap exists as previous models also comprised a depression factor and a mania factor (e.g., refs. [15, 67]). The often-reported factors positive and negative symptoms are split into all three factors in the present results while disorganization best fits the present second factor MA.

The derived lifetime psychopathological factors were used to investigate underlying GMV and CT correlates. We were able to detect numerous associations between the PHS and both GMV and CT in both temporal and frontal regions. We did not detect any interactions for both factor x diagnosis on a whole-brain level, nor in post hoc analyses of the significant clusters. These findings do not exclude that the severity of both brain structural alterations and psychopathological syndromes may vary by diagnosis. Our study aligns with previous studies proposing overlaps in acute psychopathology, brain structure as well as genetics across MDD, BD and SSD [11–13, 68]. Combining a data-driven approach to psychopathology with studying neuroanatomical and genetic correlates may help elucidate the biological underpinnings of complex syndromes in psychiatric disorders. Approaches such as those applied in the present study can reveal intra- and inter-disorder heterogeneity and thus could support the establishment of treatments specific to symptom or syndrome in the next step.

When comparing our results to previous dimensional studies, a recent study also identified subcortical volume reductions associated with hallucinations as well as delusions [69], but reductions of superior temporal areas have also been well established in SSD [1, 3, 70, 71]. The present findings are also in line with a recent investigation where psychotic symptoms were negatively correlated with CT in a large sample of SSD patients, relatives and healthy controls [72]. Consistent with previous studies in SSD, we found cortical thinning in the bilateral STG to be correlated with the PHS factor [73], indicating this brain structure to be a core feature of positive symptomatology.

Exploratory GWAS and PRS analyses suggest a contribution of common genetic variants to all three factor dimensions, supporting the hypothesis that symptoms observed in different diagnostic groups may be influenced by the same genetic variants across diagnostic boundaries [14, 29]. Interestingly, the genome-wide significant loci of our GWAS implicated protein-coding genes that both might be linked to psychiatric disorders. *ADAMTS19* is a member of the ADAMTS (a disintegrin and metalloproteinase with thrombospondin motif) family [74], which might be involved in neuroplasticity [75]. The *RAD18* gene encodes for a DNA damage repair protein [74, 76]. Notably, a study by Alsulami and colleagues provided evidence that RAD18 interacts with SETD1A [76], which has previously been associated with SSD at the rare variant level [77, 78]. As it is known that genome-wide significant lead variants do not necessarily exert their effects through the nearest genes (e.g., ref. [79]), the above discussed functional interpretations should be viewed with caution, as further bioinformatic and functional analyses are needed to identify the gene(s) relevant at the identified loci.

Finally, despite the associations at the genetic level, we did not detect an association between the MA or DEP factor and brain morphology. This suggests that even though aspects of lifetime psychopathology might at least be partially influenced by genetic factors, this might not necessarily be detectable on a neural level. It could thus be argued that a dimensional approach is even more important than a narrow nosology as these associations might be subtle and implications for translation into treatment options are not as clear, yet.

### Limitations

There are several limitations to be considered: First, as lifetime psychopathology was assessed only at one point in this study, a bias may arise in favor of symptoms that have occurred recently or are currently present, as they could be more salient in the individual’s memory. This bias could lead to an overemphasis on these symptoms during the assessment process [80]. As a result, symptoms that occurred in the past may be underreported or forgotten entirely. We tried to circumvent these biases by carefully examining every hospital record available for each patient, but these were not available for all patients included here. In addition, the used psychopathological scale did not include the full symptomatic spectrum, which restricted the identification of psychopathological factors.

Second, sample sizes of each diagnostic category were unequal. The aim of the present study was to investigate syndrome-brain structure and syndrome-genetic associations dimensionally rather than within categorical diagnoses. The presence of psychotic and manic symptoms in the MDD group might be limited, which may result in restricted variance found for the PHS factor. While results can be considered as diagnosis-shared, severity may be differing across diagnoses.

Third, MRI techniques in general might not be able to detect subtle differences in locations of effects if these occurs in close proximity. In addition, true effects between groups might be mapped onto the same neural circuit while in fact there are differences on the underlying cellular level [81].

Fourth, pharmacological treatment was considered as three dummy coded variables to account for the current intake of antidepressants, antipsychotics, and mood stabilizers. This approach does not take into account both the dosage and lifetime cumulative intake of psychotropic medication, which might have influenced our results.

Finally, the available sample size represents a limitation for the genetic analyses, as the robust detection of genetic associations with small effect sizes usually requires meta-analytical efforts involving multiple cohorts [82]. Thus, the exploratory nature of the presented GWAS should be considered in the interpretation of our findings.

## Conclusion

This study comprehensively investigated the association of lifetime psychopathological dimensions and brain morphometric markers as well as underlying genetic factors. At the level of brain imaging, GMV and CT reductions in temporal, occipital, and limbic structures were found to be correlated with paranoid-hallucinatory symptoms in a transdiagnostic sample. On the genetic level, we identified genome-wide significant loci for MA and DEP factors, as well as positive effects of specific PRS on different factors. These findings suggest that genetic factors contribute to the identified factor dimensions. The results presented in this study highlight the importance of i) dimensional modeling and ii) transdiagnostic research gaining a better understanding of pathophysiological mechanisms underlying MDD, BD and SSD.

### Supplementary information

Supplementary Material

## Data Availability

The data and code supporting the findings of this study can be accessed by contacting the corresponding author (FS).
