# Supplementary material for: Factor analysis of lifetime psychopathology and its brain morphometric and genetic correlates in a transdiagnostic sample
Source: Transl Psychiatry. 2024 Jun 3;14:235. doi: 10.1038/s41398-024-02936-6 (PMC11148082; doi:10.1038/s41398-024-02936-6)
Supplement: Supplementary file 1 — Supplementary Material [file 41398_2024_2936_MOESM1_ESM.docx]

**Supplement: Factor analysis of lifetime psychopathology and its brain morphometric and genetic correlates in a transdiagnostic sample**

**Table of content**

[eTable1: Descriptive statistics of the age- and sex matched subsample n=324 2](#_Toc165881129)

[eTable2(a-d): Results of 4 explorative factor models using bootstrapped analyses in R 3](#_Toc165881130)

[eFigure1: Scree plot of the explorative factor analyses in R using the EFAutilities package 6](#_Toc165881131)

[eResults1: Results of the confirmatory analyses of the matched sample n=324 7](#_Toc165881132)

[eResults2: Results of the confirmatory analyses within each diagnostic category 7](#_Toc165881133)

[eResults3: Comparison of factor loadings between diagnostic categories 7](#_Toc165881134)

[eFigure2: Distribution of identified factors across DSM-IV-TR categorical diagnoses 8](#_Toc165881135)

[eResults4: Interaction analyses (factor x diagnosis) on significant GMV and CT clusters in the total sample 9](#_Toc165881136)

[eResults5: Results of the GMV and CT analyses in the matched sample n=324 9](#_Toc165881137)

[eTable3: Results of the multiple regression analysis of factor 1 (paranoid-hallucinatory syndrome) and its local GMV correlates in the matched sample (n=324) on whole brain level at p<.05, FWE corrected 10](#_Toc165881138)

[eTable4: Results of the multiple regression analysis of factor 1 (paranoid-hallucinatory syndrome) and its local CT correlates in the matched sample (n=324) on whole brain level at p<.05, FWE corrected 10](#_Toc165881139)

[eTable5: Overview of the independent lead variants with p < 1e-6 in GWAS for factor 1 "paranoid-hallucinatory syndrome", factor 2 "mania" and factor 3 "depression" 34](#_Toc165881140)

[eFigure3: Post hoc visualization of the interaction analyses of the left hippocampus/amygdala GMV cluster (k=560) and factor 1 “paranoid-hallucinatory syndrome” 34](#_Toc165881141)

[eFigure4: Post hoc visualization of the interaction analyses of the right angular gyrus GMV (k=150) and factor 1 “paranoid-hallucinatory syndrome” 35](#_Toc165881142)

[eFigure5: Post hoc visualization of the interaction analyses of the right hippocampus/amygdala GMV (k=83) and factor 1 “paranoid-hallucinatory syndrome” 36](#_Toc165881143)

[eFigure6: Post hoc visualization of the interaction analyses of the right medial frontal cerebrum GMV (k=64) and factor 1 “paranoid-hallucinatory syndrome” 37](#_Toc165881144)

[eFigure7: Post hoc visualization of the interaction analyses of the left supramarginal cortex CT (k=657) and factor 1 “paranoid-hallucinatory syndrome” 38](#_Toc165881145)

[eFigure8: Post hoc visualization of the interaction analyses of the left superior temporal cortex CT (k=777) and factor 1 “paranoid-hallucinatory syndrome” 39](#_Toc165881146)

[eFigure9: Post hoc visualization of the interaction analyses of the right superior temporal cortex CT (k=236) and factor 1 “paranoid-hallucinatory syndrome” 40](#_Toc165881147)

[eFigure10: Post hoc visualization of the interaction analyses of the right lateral occipital cortex CT (k=47) and factor 1 “paranoid-hallucinatory syndrome”. 41](#_Toc165881148)

[eFigure11: Genetic associations with factor dimension "Paranoid-hallucinatory syndrome” 42](#_Toc165881149)

[eFigure12: Genetic associations with factor dimension "Mania" 43](#_Toc165881150)

[eFigure13: Genetic associations with factor dimension "Depression" 44](#_Toc165881151)

[eFigure14: PRS association analysis by diagnostic subgroups 45](#_Toc165881152)

# eTable1: Descriptive statistics of the age- and sex matched subsample n=324

|  | **Major depressive disorder**  **(n=108)** | **Bipolar disorder**  **(n=108)** | **Schizophrenia spectrums disorders**  **(n=108)** | **Group comparison**  **(F/Chi-values in brackets)** |
| --- | --- | --- | --- | --- |
| age | 37.44 (11.45) | 38.6 (11.13) | 37.47 (11.46) | *p* = .454  (.79) |
| sex | m = 57  f = 51 | m = 50  f = 58 | m = 57  f = 51 | *p* = .546  (1.21) |
| years of education | 12.88 (2.69) | 13.99 (2.92) | 12.53 (2.64) | *p* < .001^a^  (7.48) |
| age of onset | 26.75 (12.30) | 22.87 (10.24) | 22.72 (9.25) | *p =* .018^b^  (4.09) |
| TIV | 1556.69 (182.91) | 1584.11 (144.1) | 1582.81 (180.36) | *p* = .378  (.98) |

^a^ MDD < BD; SSD < BD

^b^ BD < MDD; SSD < MDD

TIV=total intracranial volume

# eTable2: Results of 4 explorative factor models using bootstrapped analyses in R

*a) two factors*

| **Factor** | **Item** | **Symptom** | **Loading** |
| --- | --- | --- | --- |
| 1 | Opcrit61 | Delusions of passivity | 0.7186302 |
|  | Opcrit64 | Delusions and hallucinations last for one week | 0.69725836 |
|  | Opcrit68 | Thought broadcast | 0.69493269 |
|  | Opcrit66 | Thought insertion | 0.68672387 |
|  | Opcrit58 | Delusions of influence | 0.6831929 |
|  | Opcrit62 | Primary delusional perception | 0.65961488 |
|  | Opcrit55 | Well organized delusions | 0.65001771 |
|  | Opcrit54 | Persecutory delusions | 0.62193331 |
|  | Opcrit60 | Widespread delusions | 0.61604386 |
|  | Opcrit74 | Running commentary voices | 0.59316838 |
|  | Opcrit73 | Third person auditory hallucinations | 0.56862342 |
|  | Opcrit59 | Bizarre delusions | 0.55298332 |
|  | Opcrit67 | Thought withdrawal | 0.55176122 |
|  | Opcrit63 | Other primary delusions | 0.53923173 |
|  | Opcrit77 | Non-affective hallucination in any modality | 0.52241137 |
| 2 | Opcrit35 | Elevated mood | 0.88125096 |
|  | Opcrit19 | Excessive activity | 0.8344525 |
|  | Opcrit30 | Pressured speech | 0.80312123 |
|  | Opcrit56 | Increased self esteem | 0.79792957 |
|  | Opcrit20 | Reckless activity | 0.77466403 |
|  | Opcrit22 | Reduced need for sleep | 0.76490046 |
|  | Opcrit31 | Thoughts racing | 0.74233794 |
|  | Opcrit21 | Distractibility | 0.54430714 |

*b) 3 factors*

| **Factor** | **Item** | **Symptom** | **Loading** |
| --- | --- | --- | --- |
| 1 | Opcrit61 | Delusions of passivity | 0.71113877 |
|  | Opcrit64 | Delusions and hallucinations last for one week | 0.69772454 |
|  | Opcrit68 | Thought broadcast | 0.69706931 |
|  | Opcrit66 | Thought insertion | 0.68144144 |
|  | Opcrit58 | Delusions of influence | 0.68113481 |
|  | Opcrit62 | Primary delusional perception | 0.65742341 |
|  | Opcrit55 | Well organized delusions | 0.65045146 |
|  | Opcrit60 | Widespread delusions | 0.62104784 |
|  | Opcrit54 | Persecutory delusions | 0.62009573 |
|  | Opcrit74 | Running commentary voices | 0.60230526 |
|  | Opcrit73 | Third person auditory hallucinations | 0.57167791 |
|  | Opcrit59 | Bizarre delusions | 0.55200674 |
|  | Opcrit67 | Thought withdrawal | 0.54968783 |
|  | Opcrit63 | Other primary delusions | 0.54319511 |
|  | Opcrit77 | Non-affective hallucination in any modality | 0.52218947 |
| 2 | Opcrit35 | Elevated mood | 0.8869237 |
|  | Opcrit19 | Excessive activity | 0.82917998 |
|  | Opcrit30 | Pressured speech | 0.80357298 |
|  | Opcrit56 | Increased self esteem | 0.79508134 |
|  | Opcrit20 | Reckless activity | 0.76533184 |
|  | Opcrit22 | Reduced need for sleep | 0.75978711 |
|  | Opcrit31 | Thoughts racing | 0.73205637 |
|  | Opcrit21 | Distractibility | 0.52453016 |
| 3 | Opcrit39 | Loss of pleasure | 0.67820048 |
|  | Opcrit25 | Loss of energy/tiredness | 0.63488745 |
|  | Opcrit37 | Dysphoria | 0.60321497 |
|  | Opcrit41 | Lack of concentration | 0.50680415 |

*c) 4 factors*

| **Factor** | **Item** | **Symptom** | **Loading** |
| --- | --- | --- | --- |
| 1 | Opcrit64 | Delusions and hallucinations last for one week | 0.7419598 |
|  | Opcrit58 | Delusions of influence | 0.67008969 |
|  | Opcrit62 | Primary delusional perception | 0.66234816 |
|  | Opcrit74 | Running commentary voices | 0.63791568 |
|  | Opcrit54 | Persecutory delusions | 0.63777479 |
|  | Opcrit60 | Widespread delusions | 0.61983275 |
|  | Opcrit73 | Third person auditory hallucinations | 0.59572571 |
|  | Opcrit55 | Well organized delusions | 0.59217412 |
|  | Opcrit68 | Thought broadcast | 0.56040415 |
|  | Opcrit75 | persecuting voices | 0.55369279 |
|  | Opcrit65 | Delusions of persecution/jealousy with hallucinations | 0.53192688 |
|  | Opcrit77 | Non-affective hallucinations of any kind | 0.51963127 |
| 2 | Opcrit35 | Elevated mood | 0.88749412 |
|  | Opcrit19 | Excessive activity | 0.83001028 |
|  | Opcrit30 | Pressured speech | 0.80389023 |
|  | Opcrit56 | Increased self esteem | 0.79443685 |
|  | Opcrit20 | Reckless activity | 0.76706152 |
|  | Opcrit22 | Reduced need for sleep | 0.75833445 |
|  | Opcrit31 | Thoughts racing | 0.73104458 |
|  | Opcrit21 | Distractibility | 0.52702932 |
| 3 | Opcrit39 | Loss of pleasure | 0.68226506 |
|  | Opcrit25 | Loss of energy/tiredness | 0.64355002 |
|  | Opcrit37 | Dysphoria | 0.60451471 |
|  | Opcrit41 | Lack of concentration | 0.50860201 |
| 4 | Opcrit66 | Thought insertion | 0.80392806 |
|  | Opcrit61 | Delusions of passivity | 0.78247893 |
|  | Opcrit67 | Thought withdrawal | 0.50656667 |

*d) 5 factors*

| **Factor** | **Item** | **Symptom** | **Loading** |
| --- | --- | --- | --- |
| 1 | Opcrit64 | Delusions and hallucinations last for one week | 0.73384835 |
|  | Opcrit58 | Delusions of influence | 0.66785487 |
|  | Opcrit62 | Primary delusional perception | 0.66204709 |
|  | Opcrit54 | Persecutory delusions | 0.63353398 |
|  | Opcrit74 | Running commentary voices | 0.63058018 |
|  | Opcrit60 | Widespread delusions | 0.62309018 |
|  | Opcrit55 | Well organized delusions | 0.59078412 |
|  | Opcrit73 | Third person auditory hallucinations | 0.58864761 |
|  | Opcrit68 | Thought broadcast | 0.56026174 |
|  | Opcrit75 | Persecuting voices | 0.54909176 |
|  | Opcrit65 | Delusions of persecution/jealousy with hallucinations | 0.52443502 |
|  | Opcrit77 | Non-affective hallucinations of any kind | 0.51634292 |
| 2 | Opcrit35 | Elevated mood | 0.88983081 |
|  | Opcrit19 | Excessive activity | 0.83197068 |
|  | Opcrit30 | Pressured speech | 0.80580084 |
|  | Opcrit56 | Increased self esteem | 0.79772686 |
|  | Opcrit20 | Reckless activity | 0.76908659 |
|  | Opcrit22 | Reduced need for sleep | 0.75989052 |
|  | Opcrit31 | Thoughts racing | 0.73369323 |
|  | Opcrit21 | Distractibility | 0.53044062 |
| 3 | Opcrit39 | Loss of pleasure | 0.6759944 |
|  | Opcrit25 | Loss of energy/tiredness | 0.63909346 |
|  | Opcrit37 | Dysphoria | 0.59216354 |
|  | Opcrit41 | Lack of concentration | 0.50044758 |
| 4 | Opcrit66 | Thought insertion | 0.81388741 |
|  | Opcrit61 | Delusions of passivity | 0.7843957 |
|  | Opcrit67 | Thought withdrawal | 0.51409649 |
| 5 | Opcrit51 | Weight increase | 0.86371274 |
|  | Opcrit50 | Increased appetite | 0.83639152 |

# eFigure1: Scree plot of the explorative factor analyses in R using the EFAutilities package


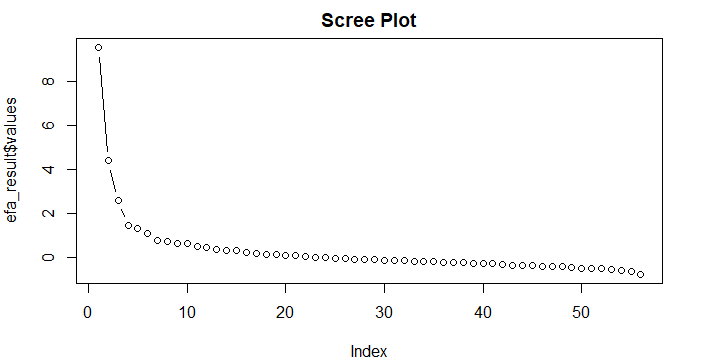


# eResults1: Results of the confirmatory analyses of the matched sample n=324

To rule out potential confounding effects of unequally distributed diagnostic categories, we performed the confirmatory factor analysis again in an age and sex matched sub-sample. The explorative assumed three-factor model revealed a good fit in the matched sample: *x*^2^=529.450, *df*=316, *p*<.0001, *CFI*=.918, *RMSEA*=.046.

# eResults2: Results of the confirmatory analyses within each diagnostic category

To rule out potential confounding effects driven by diagnostic categories, we performed the

confirmatory factor analysis in each diagnostic category. Fit values were as follows:

MDD: *x*^2^=1168.824, *df*=316, *p*<.0001, CFI=.497, RMSEA=.058.

BD: *x*^2^=668.366, *df*=291, *p*<.0001, *CFI*=.636, *RMSEA*=.101.

SSD: *x^2^*=455.26, *df*=316, *p*<.0001, *CFI*=.85, *RMSEA*=.064.

# eResults3: Comparison of factor loadings between diagnostic categories

In addition to confirmatory analyses within each diagnostic category and in an age and sex matched sub-sample, we investigated differences of the factor loadings between diagnostic categories using a non-parametric ANOVA (Kruskal-Wallis). Patients differed significantly in all three factors (PHS: *χ^2^* (2,1)=489, *p*<.001, *Ɛ²*=.472; MA: *χ^2^* (2,1)=426, *p*<.001, *Ɛ²*=.410; DEP: *χ^2^* (2,1)=121, p<.001, *Ɛ²*=.116). Post hoc comparisons revealed differences between all diagnostic categories and factors (all *p*s<.001). Factor distributions across DSM-IV-TR diagnoses are provided in *eFigure2*.

# eFigure2: Distribution of identified factors across DSM-IV-TR categorical diagnoses


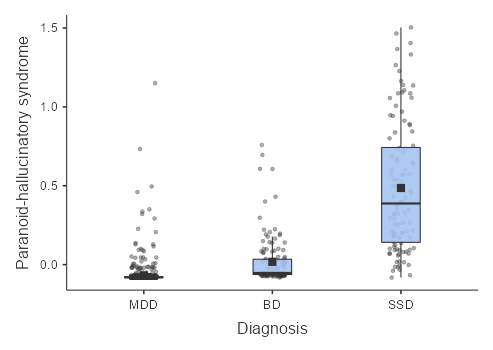


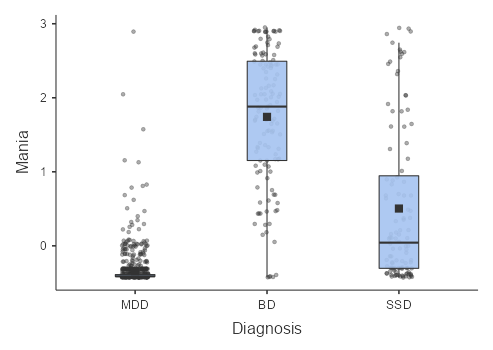


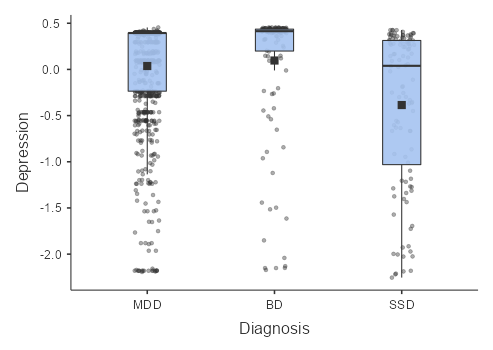


# eResults4: Interaction analyses (factor x diagnosis) on significant GMV and CT clusters in the total sample

To investigate if categorical diagnosis and lifetime psychopathological factor would interact on the identified factors, we performed interaction analyses using the “lm-function” in R. We did not identify significant interaction effects (all *p*s>0.05).

# eResults5: Results of the GMV and CT analyses in the matched sample n=324

Since DSM-IV diagnostic categories were unequally distributed in the present sample, we performed multiple regression analyses again in an age and sex matched sub-sample with same *n* per diagnosis. Therefore, we used latent, standardized factors scores drawn from the confirmatory analyses of this sub-sample. Results of the multiple regression analyses of factor 1 (paranoid-hallucinatory syndrome) and its local GMV correlates in the matched sample are presented in eTable3. Results of the CT correlates are presented in eTable4.

Furthermore, we tested if significant clusters from the whole brain analyses in the total sample could be replicated in the matched sample, too. We were able to replicate all from the whole brain analyses in the total sample: For GMV: left hippocampus/amygdala (*k*=368 voxels, *x/y/z=*-27/0/-28, *t*=3.81, *p*=.005, FWE), right angular gyrus (*k*=150 voxels, *x/y/z=*58/-64/22, *t*=5.54, *p*<.001, FWE), right amygdala (*k*=16 voxels, *x/y/z=*28/-3/-27, *t*=3.26, *p*=.001, FWE), right medial frontal cerebrum (*k*=64 voxels, *x/y/z*=2/60/-8, *t*=3.82, *p*=.005, FWE). For CT we were able to replicate both cluster of the left hemisphere: supramarginal cortex (*k*=777 voxels, *x/y/z*=-50/-37/26, *t*=3.93, *p*=.001, FWE) and superior temporal cortex (*k*=444 voxels, *x/y/z*=-49/-4/-11, *t*=3.27, *p*=.008, FWE). Clusters of the right hemisphere could be replicated in the matched sample as well: superior temporal cortex (*k*=196 voxels, *x/y/z*=48/10/-27, *t*=3.15, *p*=.005, FWE) and lateral occipital cortex cortex (*k*=47 voxels, *x/y/z*=46/-72/4, *t*=3.76, *p*=.001, FWE).

Interaction analyses of psychopathological factor x DSM-IV diagnostic category showed no significant interaction effects for both the whole brain and the ROI analyses.

# eTable3: Results of the multiple regression analysis of factor 1 (paranoid-hallucinatory syndrome) and its local GMV correlates in the matched sample (n=324) on whole brain level at p<.05, FWE corrected

|  |  | **MNI Coordinates** | | |  |  |
| --- | --- | --- | --- | --- | --- | --- |
| **Anatomical region** | **H** | **X** | **Y** | **Z** | **t-value** | **Cluster size** |
| **Factor I: Paranoid-hallucinatory syndrome: grey matter volume** |  |  |  |  |  |  |
|  |  |  |  |  |  |  |
| Middle occipital gyrus, angular gyrus, inferior occipital gyrus | R | 58 | -64.5 | -22.5 | 5.41 | 231 |

Only negative correlations are reported as no positive correlations were detected. H=hemisphere (L=left, R = right)

# eTable4: Results of the multiple regression analysis of factor 1 (paranoid-hallucinatory syndrome) and its local CT correlates in the matched sample (n=324) on whole brain level at p<.05, FWE corrected

|  |  | **MNI Coordinates** | | |  |  |
| --- | --- | --- | --- | --- | --- | --- |
| **Anatomical region** | **H** | **X** | **Y** | **Z** | **t-value** | **Cluster size** |
| **Factor I: Paranoid-hallucinatory syndrome: grey matter volume** |  |  |  |  |  |  |
|  |  |  |  |  |  |  |
| Precentral cortex, pars opcerularis | L | -45 | 6 | 3 | 4.23 | 232 |
|  |  |  |  |  |  |  |
| Supramarginal cortex | L | -51 | -37 | 26 | 4.05 | 114 |
|  |  |  |  |  |  |  |
| Lateral occipital cortex | R | 16 | -101 | -7 | 4.44 | 293 |

Only negative correlations are reported as no positive correlations were detected. H=hemisphere (L=left, R=right)

# *eTable5: Overview of the independent lead variants with p < 1e-6 in GWAS for factor 1* "paranoid-hallucinatory syndrome", factor 2 "mania" and factor 3 "depression"

| **Factor** | **CHR** | **SNP** | **BP** | **A1** | **A2** | **FRQ** | **INFO** | **BETA** | **SE** | **P** |
| --- | --- | --- | --- | --- | --- | --- | --- | --- | --- | --- |
| Factor1 | 1 | rs1722711 | 48672337 | T | C | 0.0783 | 0.9579 | 0.438 | 0.0868 | 5.357e-07 |
| Factor1 | 9 | rs147278663 | 107677463 | T | C | 0.9898 | 0.9235 | -1.2908 | 0.2349 | 5.028e-08 |
| Factor1 | 15 | rs4775462 | 62388058 | A | G | 0.7943 | 0.9694 | -0.2848 | 0.0572 | 7.542e-07 |
| Factor2 | 5 | rs10062519 | 128998897 | A | G | 0.9087 | 0.9626 | -0.4602 | 0.0798 | 1.1e-08 |
| Factor2 | 11 | rs9326286 | 126666201 | T | G | 0.5722 | 1.0058 | -0.2397 | 0.0457 | 1.891e-07 |
| Factor2 | 13 | rs9594624 | 42313317 | C | T | 0.6732 | 0.961 | 0.2651 | 0.0496 | 1.128e-07 |
| Factor3 | 3 | rs11131155 | 8999518 | A | T | 0.8845 | 0.9439 | 0.4053 | 0.0733 | 4.121e-08 |

eFigure3: Post hoc visualization of the interaction analyses of the left hippocampus/amygdala GMV cluster (k=560) and factor 1 “paranoid-hallucinatory syndrome”*. No interaction of categorical diagnosis and factor 1 was not present (p=.468)*

*
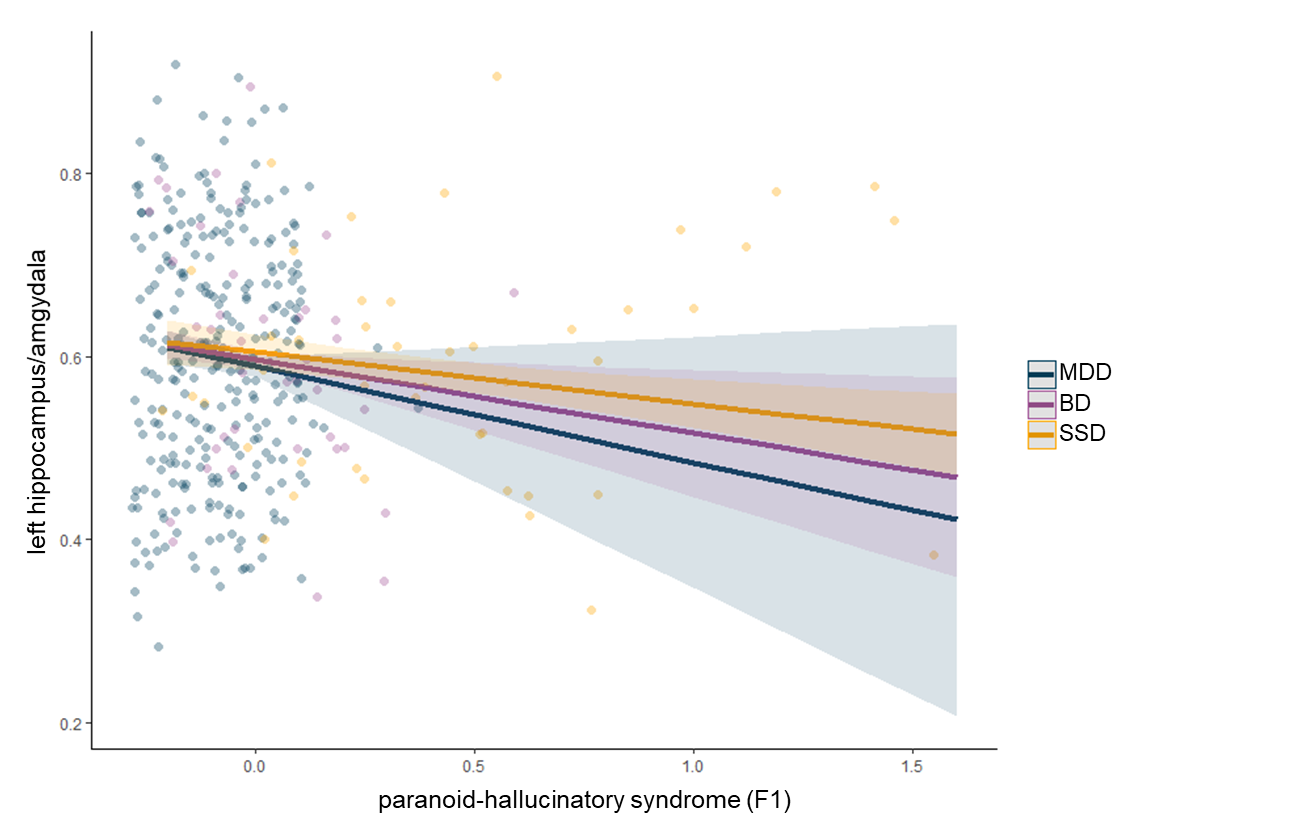
*

eFigure4: Post hoc visualization of the interaction analyses of the right angular gyrus GMV (k=150) and factor 1 “paranoid-hallucinatory syndrome”*. An interaction of categorical diagnosis and factor 1 was not present (p=.516)*

*
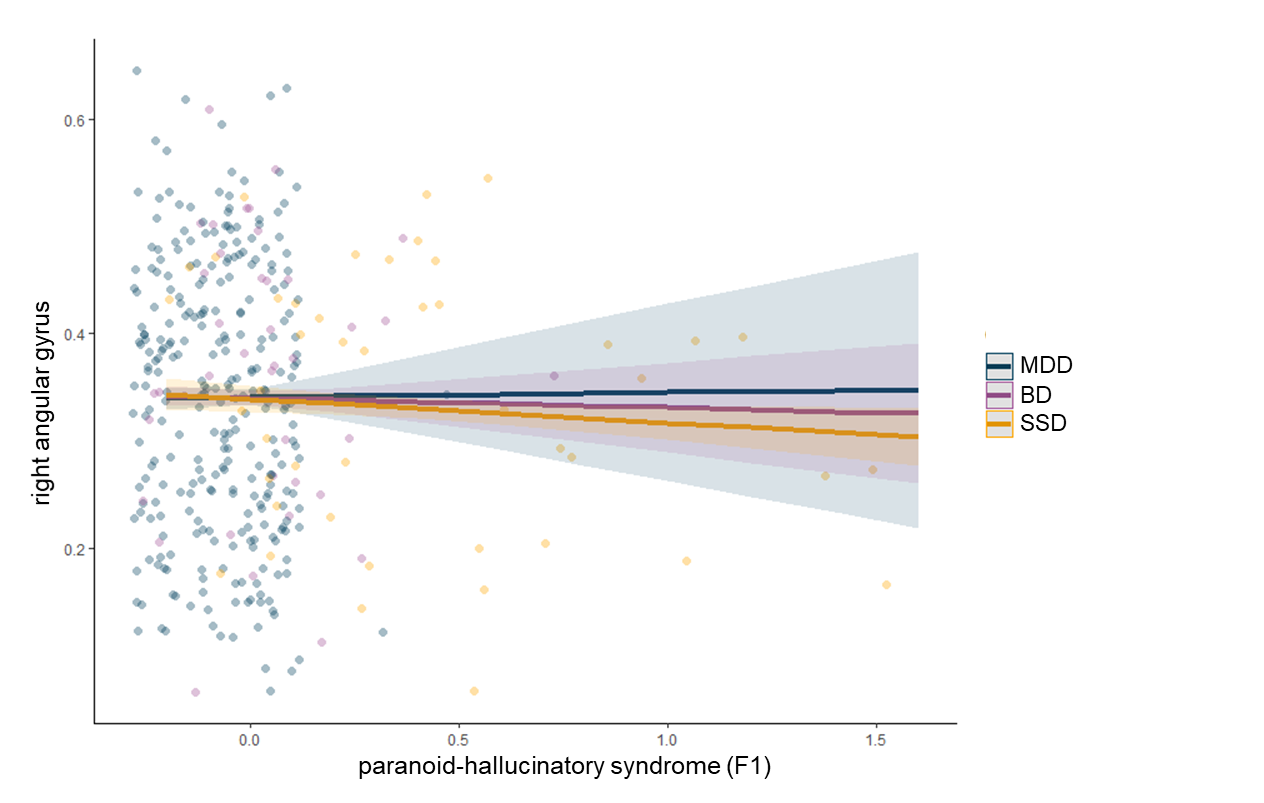
*

eFigure5: Post hoc visualization of the interaction analyses of the right hippocampus/amygdala GMV (k=83) and factor 1 “paranoid-hallucinatory syndrome”*. No interaction of categorical diagnosis and factor 1 was not present (p=.458)*

*
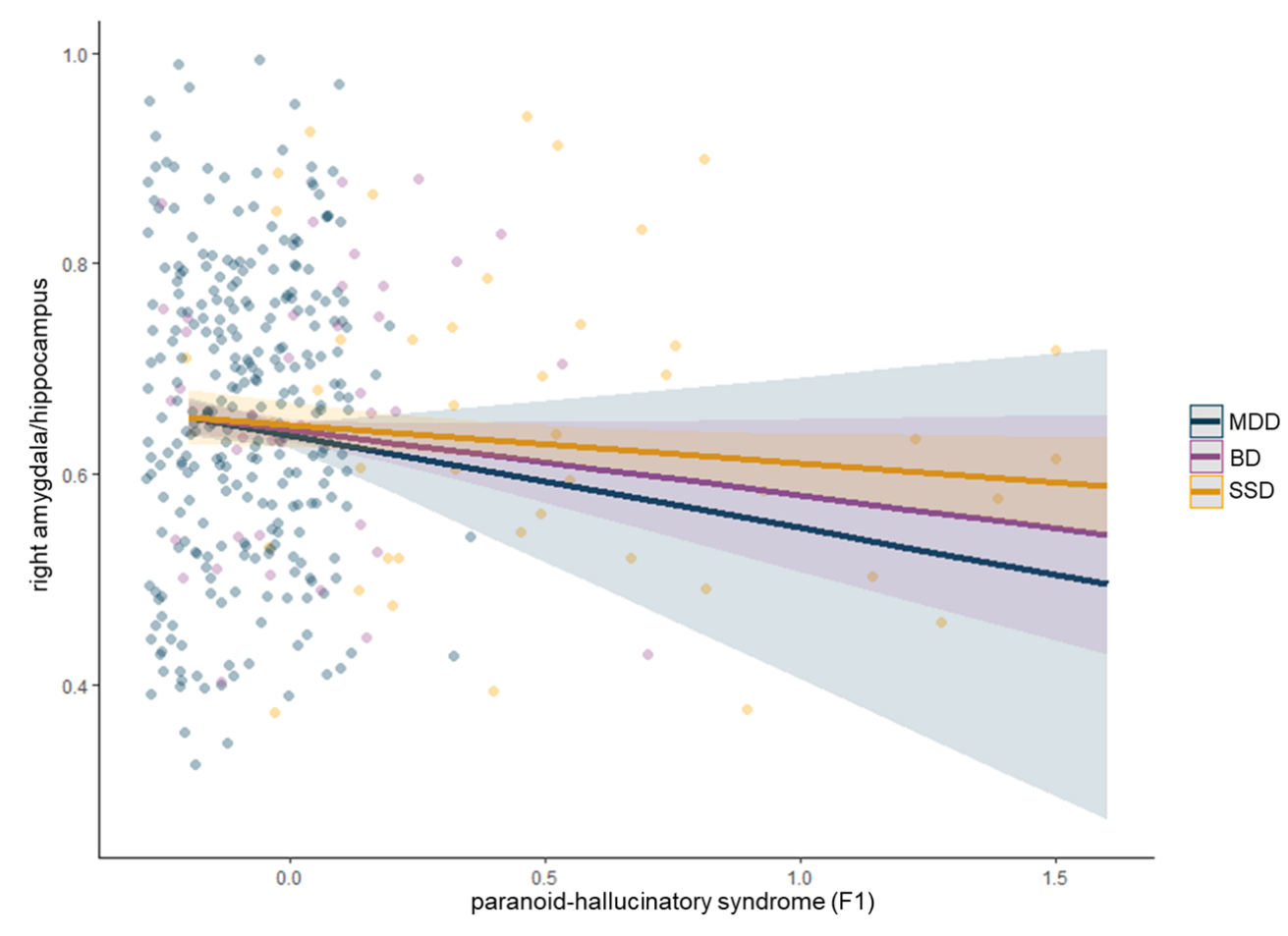
*

eFigure6: Post hoc visualization of the interaction analyses of the right medial frontal cerebrum GMV (k=64) and factor 1 “paranoid-hallucinatory syndrome”*. No interaction of categorical diagnosis and factor 1 was not present (p=.466)*

*
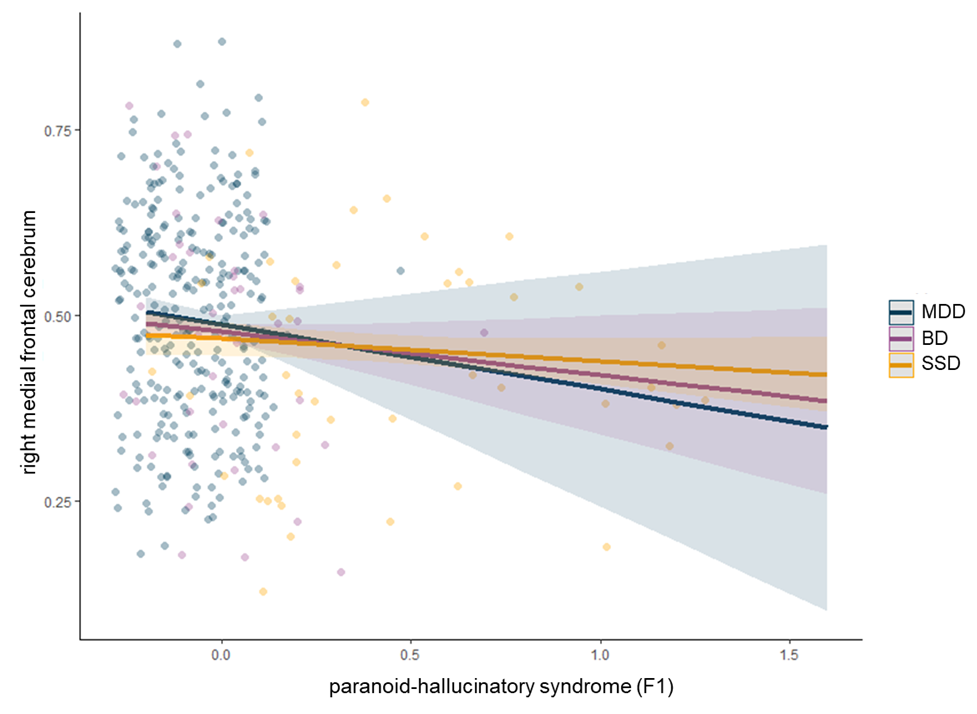
*

eFigure7: Post hoc visualization of the interaction analyses of the left supramarginal cortex CT (k=657) and factor 1 “paranoid-hallucinatory syndrome”*. An interaction of categorical diagnosis and factor 1 was not present (p=.203)*

*
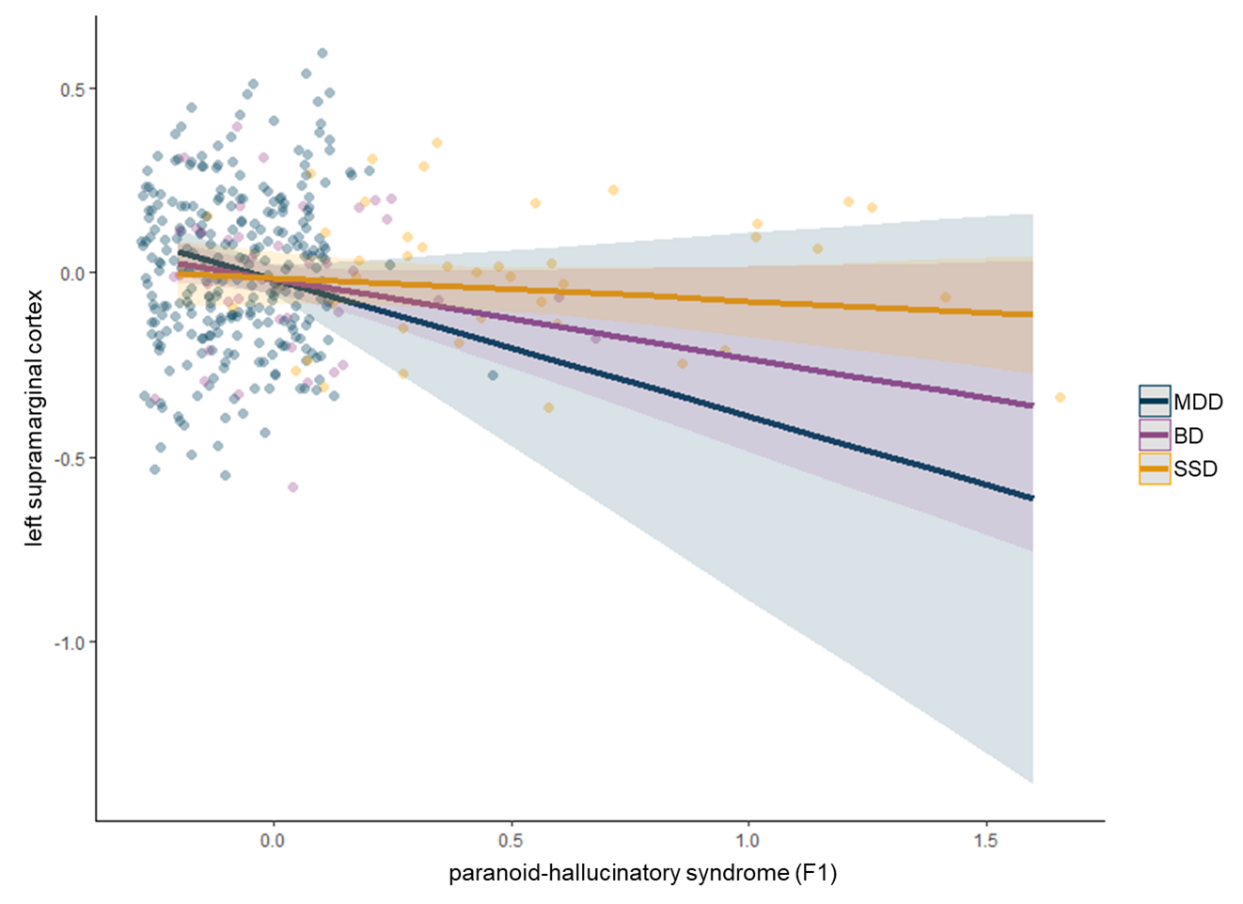
*

eFigure8: Post hoc visualization of the interaction analyses of the left superior temporal cortex CT (k=777) and factor 1 “paranoid-hallucinatory syndrome”*. An interaction of categorical diagnosis and factor 1 was not present (p=.111)*

*
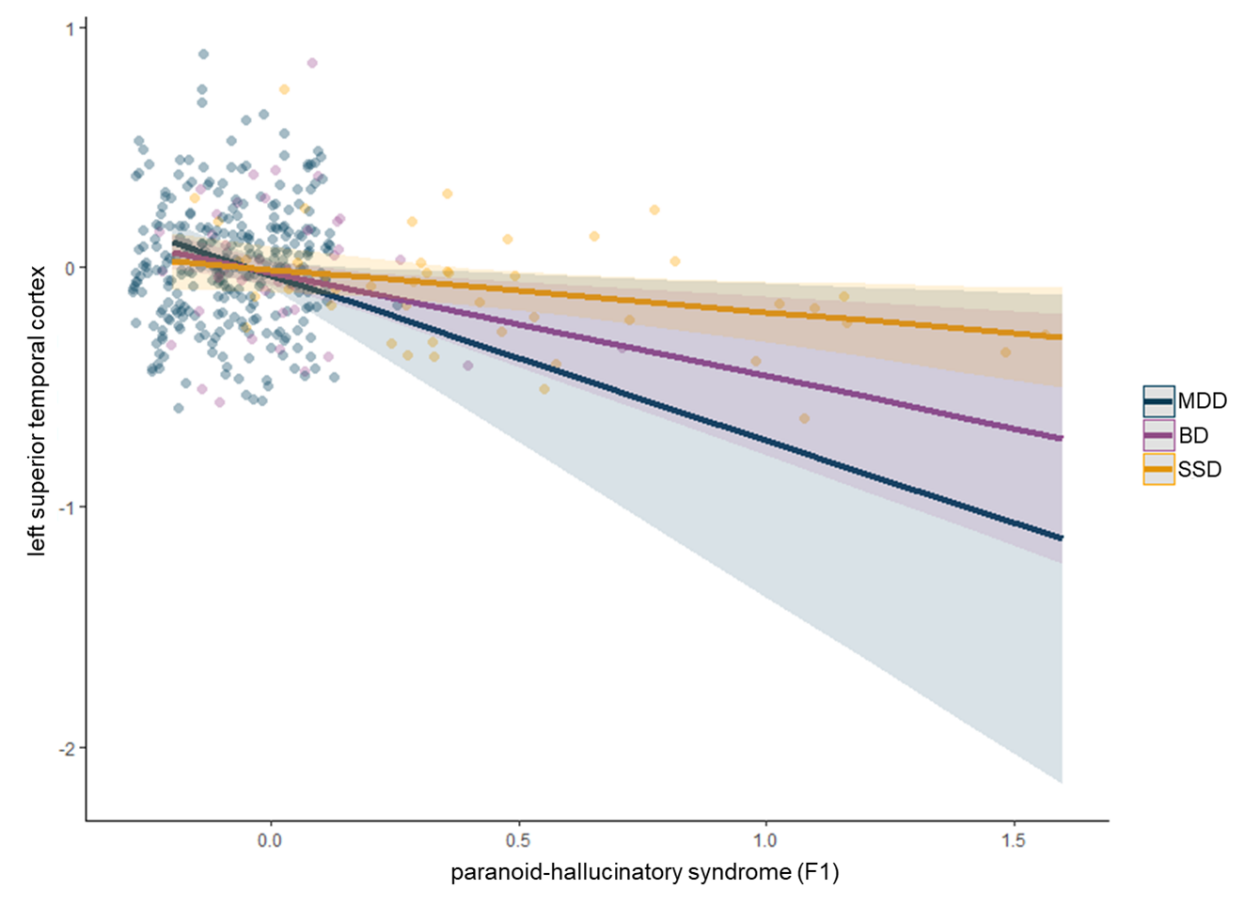
*

eFigure9: Post hoc visualization of the interaction analyses of the right superior temporal cortex CT (k=236) and factor 1 “paranoid-hallucinatory syndrome”*. An interaction of categorical diagnosis and factor 1 was present (p=.134)*

*
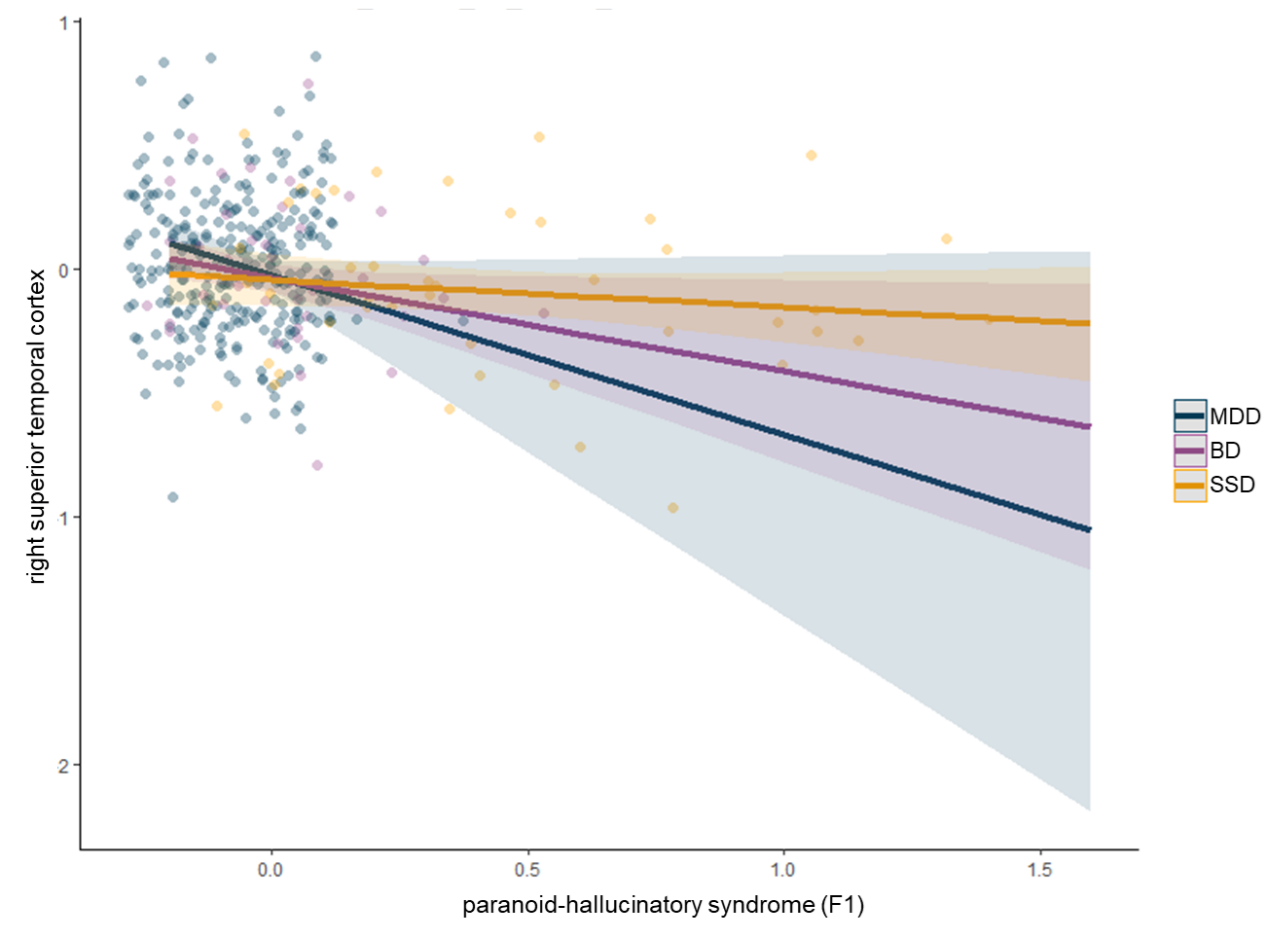
*

eFigure10: Post hoc visualization of the interaction analyses of the right lateral occipital cortex CT (k=47) and factor 1 “paranoid-hallucinatory syndrome”. *An interaction of categorical diagnosis and factor 1 was present (p=.667)*

*
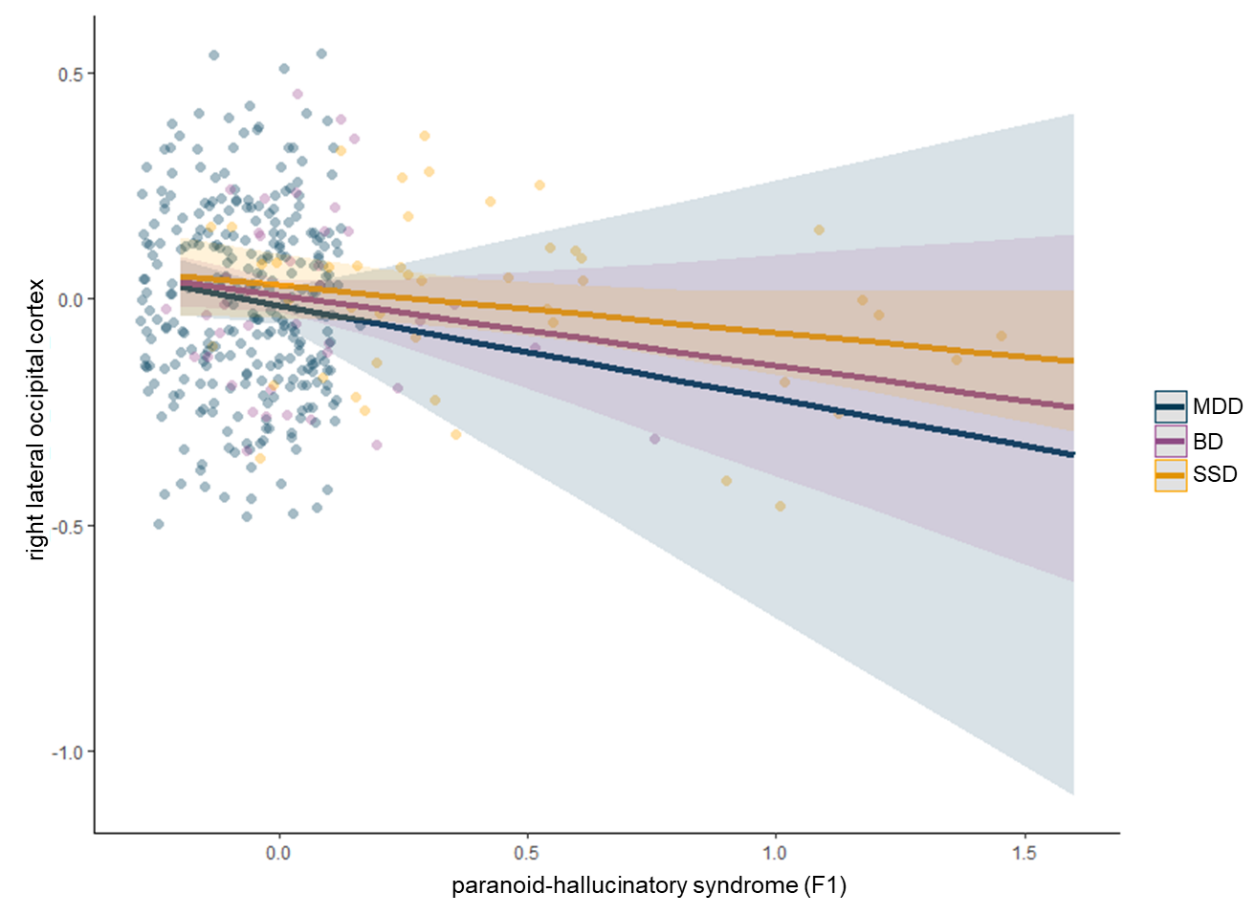
*

# eFigure11: Genetic associations with factor dimension "Paranoid-hallucinatory syndrome”

*(A) The qq-plot shows the relation between observed and expected p-values. The genomic inflation factor lambda is 1.01. (B) In the Manhattan plot, the genome-wide significance threshold (5e-8) is indicated by a red line and suggestive significance threshold (1e-6) is indicated by a blue line. Lead SNPs within at least suggestively associated loci are marked in green. p, p-value.*

*
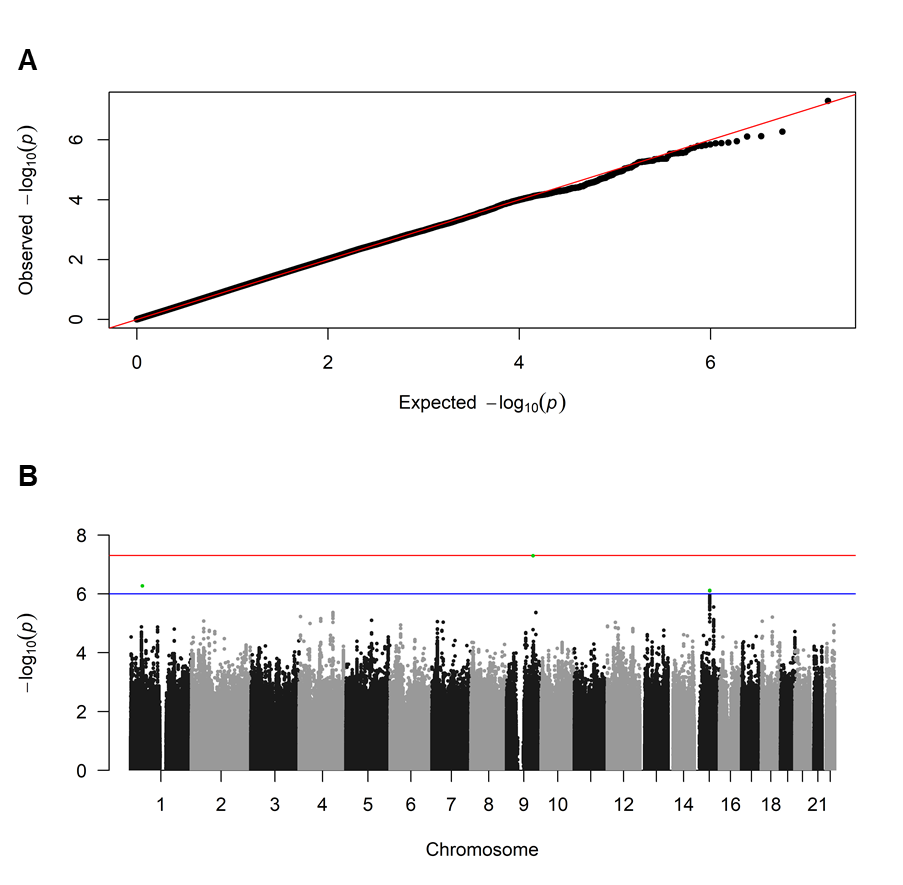
*

# eFigure12: Genetic associations with factor dimension "Mania"

*(A) The qq-plot shows the relation between observed and expected p-values. The genomic inflation factor lambda is 1.00. (B) In the Manhattan plot, the genome-wide significance threshold (5e-8) is indicated by a red line and suggestive significance threshold (1e-6) is indicated by a blue line. Lead SNPs within at least suggestively associated loci are marked in green. p, p-value.*


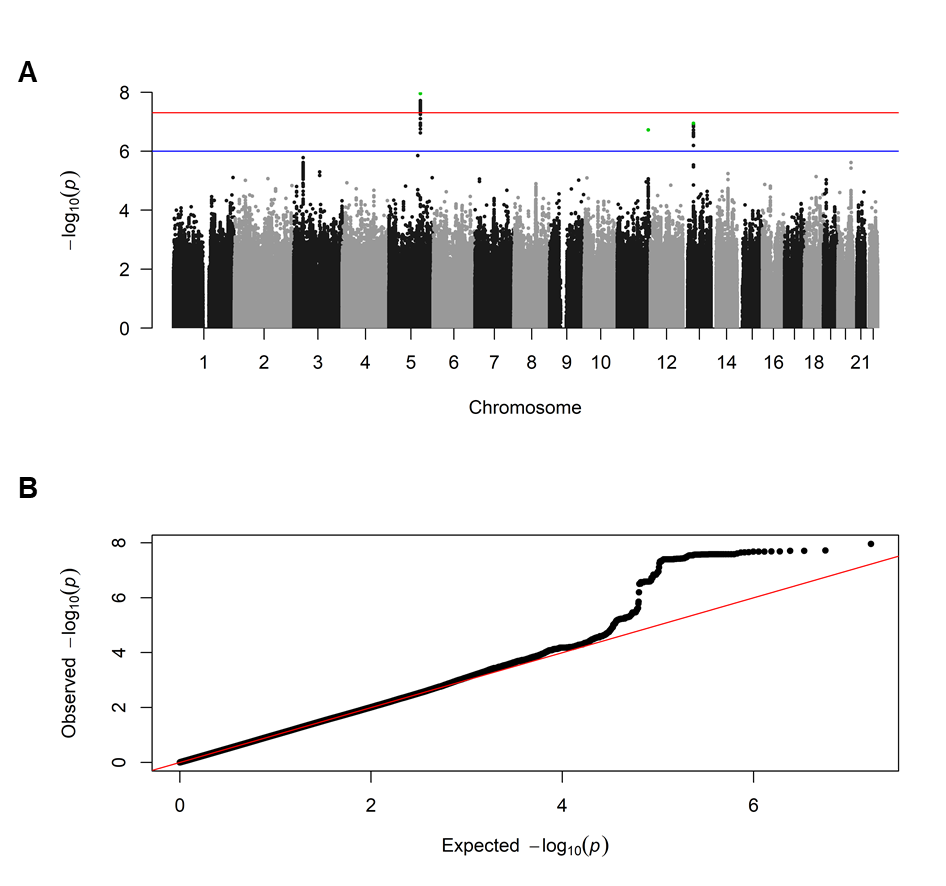


# eFigure13: Genetic associations with factor dimension "Depression"

*(A) The qq-plot shows the relation between observed and expected p-values. The genomic inflation factor lambda is 1.01. (B) In the Manhattan plot, the genome-wide significance threshold (5e-8) is indicated by a red line and suggestive significance threshold (1e-6) is indicated by a blue line. Lead SNPs within at least suggestively associated loci are marked in green. p, p-value.*

*
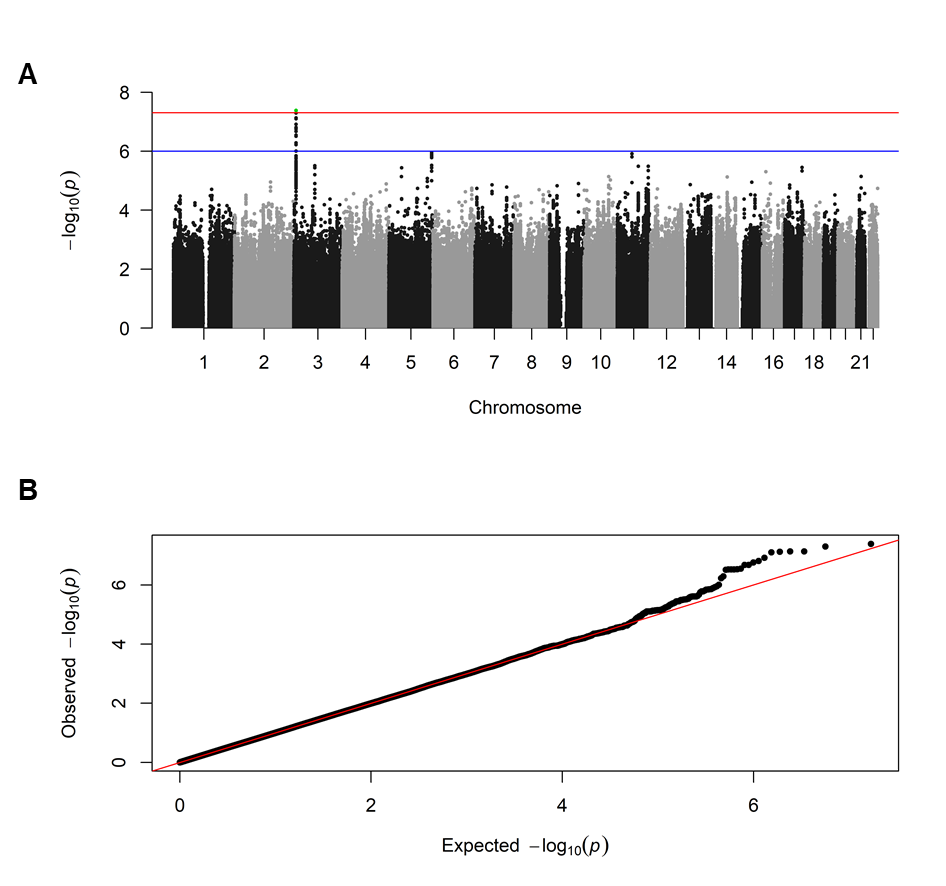
*

# eFigure14: PRS association analysis by diagnostic subgroups

*Regression of the three factors on the PRS for MDD, BD, and SZ within the complete study sample ("All") as well as the individual diagnostic subgroups is shown. BD, bipolar disorder; MDD, major depressive disorder; SSD, schizophrenia spectrum disorders; SZ, schizophrenia.*

*
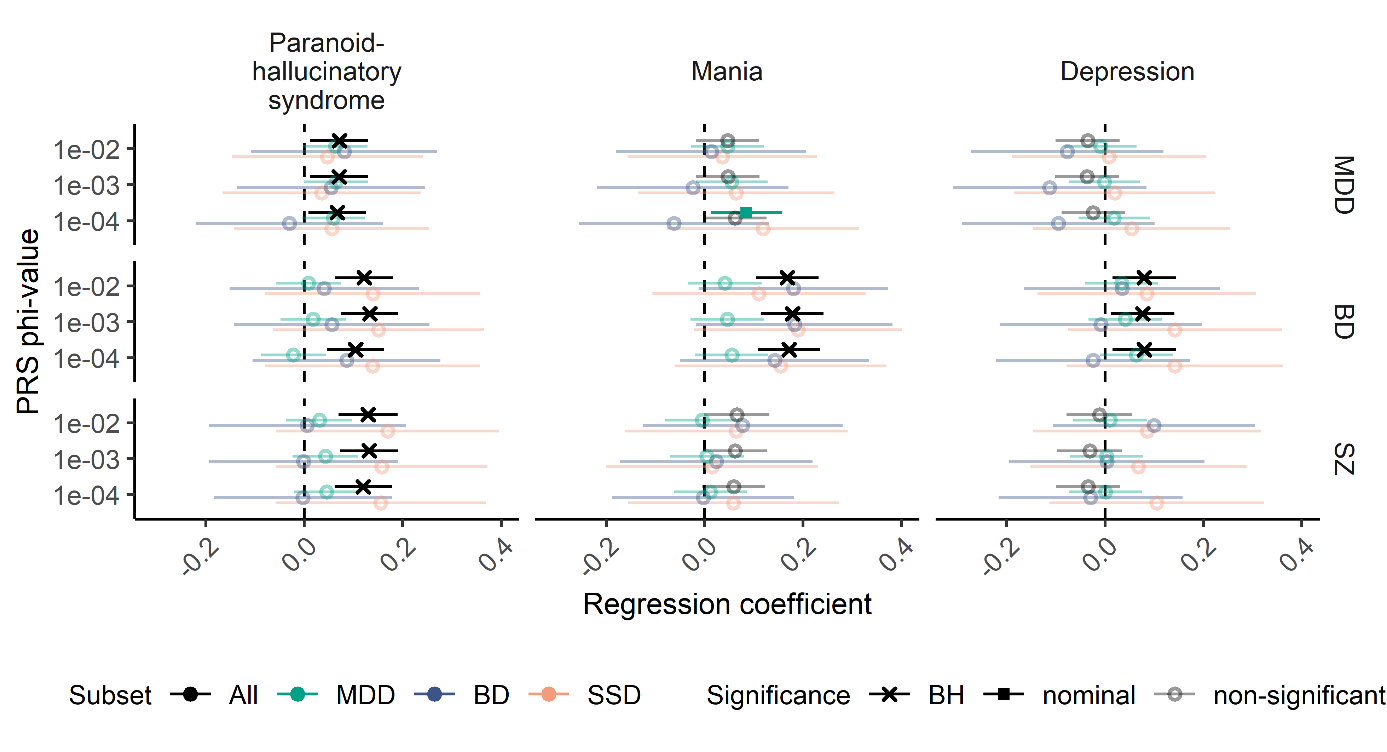
*
